# Machine Learning–Based Calibration and Performance Evaluation of Low-Cost Internet of Things Air Quality Sensors

**DOI:** 10.3390/s25103183

**Published:** 2025-05-19

**Authors:** Mehmet Taştan

**Affiliations:** Department of Electronics and Automation, Manisa Celal Bayar University, 45030 Manisa, Turkey; mehmet.tastan@cbu.edu.tr

**Keywords:** low-cost sensor, internet of things, indoor air quality, machine learning, sensor calibration

## Abstract

Low-cost air quality sensors (LCSs) are increasingly being used in environmental monitoring due to their affordability and portability. However, their sensitivity to environmental factors can lead to measurement inaccuracies, necessitating effective calibration methods to enhance their reliability. In this study, an Internet of Things (IoT)-based air quality monitoring system was developed and tested using the most commonly preferred sensor types for air quality measurement: fine particulate matter (PM_2.5_), carbon dioxide (CO_2_), temperature, and humidity sensors. To improve sensor accuracy, eight different machine learning (ML) algorithms were applied: Decision Tree (DT), Linear Regression (LR), Random Forest (RF), k-Nearest Neighbors (kNN), AdaBoost (AB), Gradient Boosting (GB), Support Vector Machines (SVM), and Stochastic Gradient Descent (SGD). Sensor performance was evaluated by comparing measurements with a reference device, and the best-performing ML model was determined for each sensor. The results indicate that GB and kNN achieved the highest accuracy. For CO_2_ sensor calibration, GB achieved R^2^ = 0.970, RMSE = 0.442, and MAE = 0.282, providing the lowest error rates. For the PM_2.5_ sensor, kNN delivered the most successful results, with R^2^ = 0.970, RMSE = 2.123, and MAE = 0.842. Additionally, for temperature and humidity sensors, GB demonstrated the highest accuracy with the lowest error values (R^2^ = 0.976, RMSE = 2.284). These findings demonstrate that, by identifying suitable ML methods, ML-based calibration techniques can significantly enhance the accuracy of LCSs. Consequently, they offer a viable and cost-effective alternative to traditional high-cost air quality monitoring systems. Future studies should focus on long-term data collection, testing under diverse environmental conditions, and integrating additional sensor types to further advance this field.

## 1. Introduction

Air pollution is widely recognized as one of the most significant environmental threats to global public health. According to the World Health Organization (WHO), 99% of the world’s population is exposed to polluted air, contributing to approximately 7 million premature deaths annually [1]. Among the key pollutants, PM_2.5_ and CO_2_ are particularly concerning due to their microscopic size, long atmospheric residence time, and chronic health impacts. Long-term exposure to these pollutants can lead to cardiovascular diseases, asthma, and respiratory complications, many of which are not immediately detectable [2,3]. Therefore, continuous and accurate air quality monitoring is vital for assessing pollutant levels and implementing effective mitigation strategies [4]. Indoor air pollution is an especially pressing concern, as individuals spend nearly 90% of their time indoors, where pollutant concentrations are often two to four times higher than outdoors [5]. Empirical studies have shown that indoor PM_2.5_ levels are directly associated with respiratory and cardiovascular health risks [6]. However, traditional reference-grade air quality monitoring stations, while highly accurate, are costly and typically limited in spatial coverage, especially in low- and middle-income regions. In response to these limitations, LCSs have emerged as practical alternatives due to their affordability, compact form factor, and ease of integration [7]. These sensors enable the creation of dense monitoring networks capable of capturing fine-scale variations in air quality over time and space [8]. LCSs are increasingly being deployed in both research and policy-driven applications to enhance environmental monitoring capabilities.

The integration of LCS into IoT frameworks further amplifies their utility. IoT-based systems allow for real-time data acquisition, wireless communication, cloud-based storage, and remote accessibility [9]. This combination enables intelligent air quality monitoring platforms that are scalable, cost-effective, and suitable for urban, industrial, and residential environments.

Despite their benefits, LCS devices face challenges in terms of measurement accuracy and environmental sensitivity, particularly to factors such as temperature and humidity, which can significantly affect sensor stability and data reliability [10]. To address these limitations, calibration methods are essential. While traditional parametric models have been used for sensor correction [11], ML algorithms have recently gained prominence due to their ability to model nonlinear relationships and improve calibration accuracy using large, complex datasets [12,13]. A variety of ML approaches, such as artificial neural network (ANN) [14], SVM [15], kNN [16], RF [17], and DT [18], have shown promising results in the context of both indoor and outdoor air quality calibration. Additionally, the reliability of LCS outputs can be further enhanced through data preprocessing techniques such as anomaly detection, missing data imputation, and smoothing. These techniques address common issues such as noise, drift, and cross-sensitivity, which frequently occur in real-world sensing environments [19,20].

The main contributions of this study are summarized as follows:

- A low-cost, IoT-based air quality monitoring system was developed using PM_2.5_, CO_2_, temperature, and humidity sensors, enabling real-time data acquisition with one-minute resolution.
- Eight machine learning algorithms were systematically evaluated to identify the most suitable calibration model for each pollutant, improving accuracy over raw sensor outputs.
- The system supports high-frequency, continuous data collection, enabling fine-grained temporal analysis of air quality trends.
- Real-time monitoring facilitates rapid detection of pollution events and raises environmental awareness.
- Indoor calibration robustness was enhanced through data collected from real emission sources, including cigarette smoke, human respiration, cooking activities, perfumes, and cleaning agents.

The remainder of this paper is organized as follows. Section 2 presents the ML methods used for sensor calibration. Section 3 introduces the developed IoT-based system and its hardware architecture. Section 4 discusses experimental results and model performances. Section 5 concludes the study and outlines recommendations for future work.

## 2. Related Works

LCSs have emerged as practical tools in regions where the deployment of reference-grade air monitoring stations is limited due to financial and infrastructural constraints. Their compact size, affordability, and ability to interface with other environmental sensors (e.g., temperature, humidity, pressure) make them adaptable for a wide range of applications [21]. Additionally, real-time data transfer via mobile or cloud-based applications enhances their utility in both residential and public monitoring settings. LCSs are extensively used to measure pollutants such as CO_2_, volatile organic compounds (VOCs), nitrogen dioxide (NO_2_), sulfur dioxide (SO_2_), and ozone (O_3_), serving as key data sources in IoT-based environmental monitoring systems [22]. However, LCSs suffer from performance limitations due to sensitivity to environmental factors. Therefore, calibration is essential to ensure data reliability and long-term accuracy. In a study that calibrated the Plantower PMS 5003 sensor using nine different ML regression algorithms, the kNN, RF, and GB models were identified as the most effective for LCS calibration, significantly enhancing sensor accuracy and providing more reliable measurements [23]. In another study, multiple linear regression (MLR), kNN, RF, and GB algorithms were evaluated for calibration of the APT Maxima sensor. The study found that the physics-based model, which accounted for the hygroscopic growth parameter, outperformed other ML methods in terms of accuracy [24]. Similarly, in a study utilizing a least-squares regression (LFR) algorithm, R^2^ values greater than 0.87 for CO_2_ and 0.73 for methane (CH_4_) were achieved, further demonstrating the high accuracy potential of LCSs when properly calibrated [25]. The HypeAIR project, which tested two real-time calibration methods––vector regression (VR) with support vector regression (SVR) and long short-term memory (LSTM) networks––over 21 months with 12 different sensors revealed that these methods outperformed both manufacturer calibrations and RF variations [26]. Another innovative approach used multilayer perceptron (MLP) structures within feedforward artificial neural networks (ANNs) to calibrate low-cost PM sensors. This method achieved high correlation values between predicted sensor data and environmental parameters (PM_1_: 0.86, PM_2.5_: 0.88, PM_10_: 0.72), underscoring the importance of factoring environmental variables into LCS calibration [27]. Symbolic regression models have also been reported to be more effective, particularly for small datasets, compared to deep neural networks (DNN) and conventional ML techniques. In cases with fewer than 9000 data points, symbolic regression exhibited superior performance [28].

Furthermore, in a study where LCS consistently underestimated pollutant concentrations in classroom settings, boosting and SVR models provided high accuracy for PM sensors. However, the study emphasized the importance of considering environmental conditions and sensor age when developing calibration models [29]. In an investigation of four PM sensors (Sharp, Honeywell, Plantower, Sensirion SPS30), ANNs were found to deliver the most accurate results, with the Sensirion SPS30 sensor exhibiting the best performance at all stages of the analysis. The study highlighted the inadequacy of factory calibrations and the necessity of recalibrating sensors before use [30]. Another study, which noted that LCSs typically recorded higher concentrations of PM_2.5_ and PM_10_, found that RF and XGBoost models outperformed others, with SHAP analysis revealing that relative humidity levels had a significant impact on model performance [31]. In the calibration of low-cost PM sensors, MLP models incorporating environmental parameters such as temperature, humidity, and pressure achieved high correlation coefficients (0.86 for PM_1_, 0.88 for PM_2.5_, and 0.76 for PM_10_), with root mean square error (RMSE) values calculated as 3.1, 4.1, and 4.9 µg/m^3^, respectively [32]. Additionally, a study exhibited that dust events could enhance the accuracy of PM measurements using LCSs, with R^2^ values of 0.92 for PM2.5 and 0.94 for PM1. Preprocessing using the Schmitz method contributed to achieving high accuracy in PM measurements [33]. Regarding CO_2_ sensors, the MH-Z19B sensor was calibrated using ML algorithms such as LSTM, LR, and RF, with the RF model achieving the highest accuracy (R^2^ = 0.97) when evaluated over different periods (5, 30, and 60 min) [34]. In a separate study, the MH-Z19B CO_2_ sensor was calibrated using the Stacking ML method, which improved sensor accuracy and achieved low error rates, including mean square error (MSE = 0.014), RMSE = 0.121, and mean absolute error (MAE = 0.031), providing a reliable calibration approach [35].

## 3. Materials and Methods

In this study, various air quality parameters, including CO_2_, PM_2.5_, relative humidity, and temperature, were measured using an IoT-based air quality monitoring system equipped with LCS. The data were processed by a 32-bit ESP8266-12E microcontroller (Espressif Systems, Shanghai, China) integrated with a WiFi module and transmitted to a cloud-based server via the Blynk platform. The Blynk platform, version 2.0, facilitated real-time monitoring and data storage, with measurements recorded at one-minute, hourly, and daily intervals. The recorded data were exported in CSV format, complete with date and time stamps, and were accessible through both web and mobile interfaces. The collected sensor data were analyzed using the Orange Data Mining software, version 3.37. During the analysis, eight different ML algorithms were applied to identify the most suitable calibration model for each air quality parameter. These algorithms included commonly used methods such as DT, LR, RF, kNN, AB, GB, SVM, and SGD.

### 3.1. Data Aquations

In the calibration of LCSs, the frequency of data acquisition plays a crucial role in the accuracy and reliability of the calibration model. Data acquisition intervals should be carefully selected based on the environmental conditions where the sensors are located, the characteristics of the target pollutants, and the specific requirements of the calibration process. LCS calibration is typically performed using field calibration methods. In this approach, sensors operate alongside reference measurement devices in their deployment areas, and sensor data are compared to reference data to adjust their accuracy. Careful planning of data acquisition frequency is essential for improving sensor accuracy and consistency. Shorter acquisition intervals (e.g., every 1–2 min) provide an advantage in capturing rapid environmental changes, enhancing the sensitivity of the calibration model and enabling more accurate predictions. The duration of the calibration period is another key factor influencing measurement accuracy. Extending calibration data acquisition over longer periods, ranging from several days to months, establishes a stronger foundation for model accuracy [36]. To optimize LCS performance and obtain reliable data, the development of appropriate calibration protocols is essential. These protocols enhance the sensors’ sensitivity to changing environmental conditions, ensuring more consistent and reliable results in air quality monitoring applications [37].

### 3.2. Reference Instrument

The Dienmern [38,39,40] DM72b (Langdeli Technology Co., Ltd., Shenzhen, China) is an IoT-based multifunctional air quality monitor built on the Tuya infrastructure, enabling real-time monitoring of various air pollutants. Tuya, version 6.5.0, is a globally popular platform providing smart home automation and IoT solutions [41]. The Dienmern DM72b is equipped with integrated sensors capable of measuring a wide range of air quality parameters, including PM_2.5_, PM_1_, PM_10_, CO_2_, formaldehyde (HCHO), total volatile organic compounds (TVOCs), temperature, and humidity. The device features automatic calibration and can trigger alarms in response to severe pollution events. With a 5 s sampling interval, it offers fast and accurate measurements. The collected air quality data are stored on a cloud server connected to the Tuya platform, allowing for retrospective access.

### 3.3. Data Preprocessing

When dividing time-dependent sensor data into training and test sets, a specific strategy must be applied due to the time series characteristics. One commonly recommended strategy in the literature for time series data is to split the data chronologically, using past data as the training set and future data as the test set. This approach allows for more accurate evaluation of the model’s ability to predict future values based on historical data. Especially in datasets with time-dependent characteristics, such as sensor data, this method provides a realistic assessment of model performance. In this study, data collected from the sensors at 5 s intervals for calibration of LCSs were transmitted to a cloud server and recorded as 1 min averages. A total of 5760 data points were collected for each sensor over a four-day period, with 75% of the data used for training the model and the remaining 25% used to evaluate model performance [42,43]. This data split was designed to enhance model accuracy and ensure the reliability of the calibration process.

**Figure 1:**
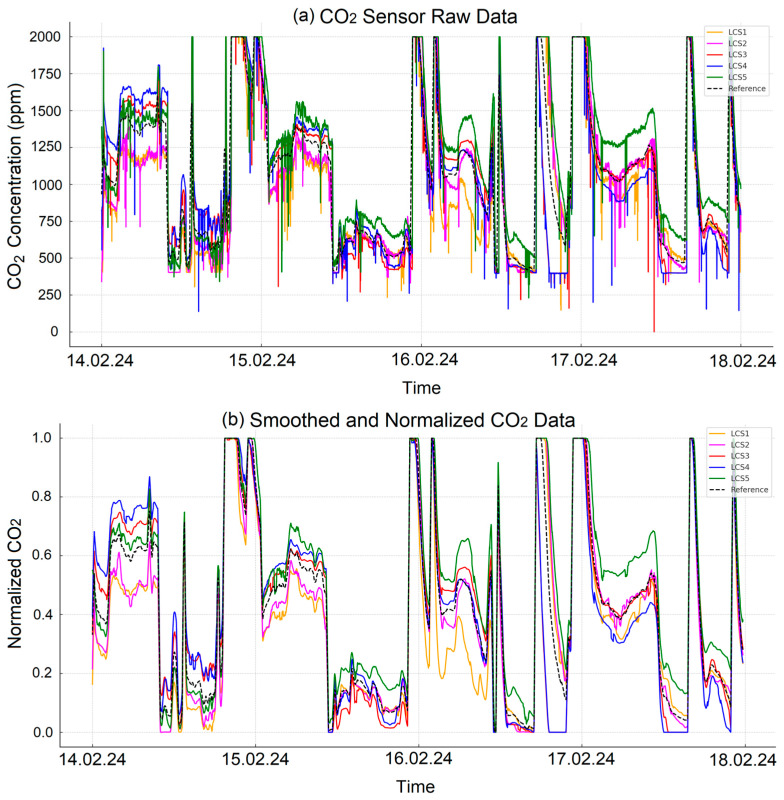
(**a**) Raw CO_2_ sensor data and (**b**) normalized and smoothed CO_2_ data.

Figure 1a illustrates the raw CO_2_ data collected from sensors, which inherently contain noise and measurement errors. Using raw data without preprocessing can significantly affect the accuracy and reliability of ML algorithms. Therefore, preprocessing is crucial for improving data quality and ensuring reliable analysis.

To address this, a moving average method with a sliding window of 10 observations was applied. This technique effectively reduces high-frequency noise and measurement errors, stabilizing the data and eliminating short-term fluctuations. Such stabilization enhances the ability of ML algorithms to produce consistent and reliable models. Following the smoothing process, normalization was performed to scale the data between 0 and 1. Normalization is a fundamental preprocessing technique that minimizes differences caused by sensors operating on varying scales, ensuring compatibility and improving the learning efficiency of ML models. Figure 1b shows the normalized and smoothed data, demonstrating reduced noise and improved stability.

**Figure 2:**
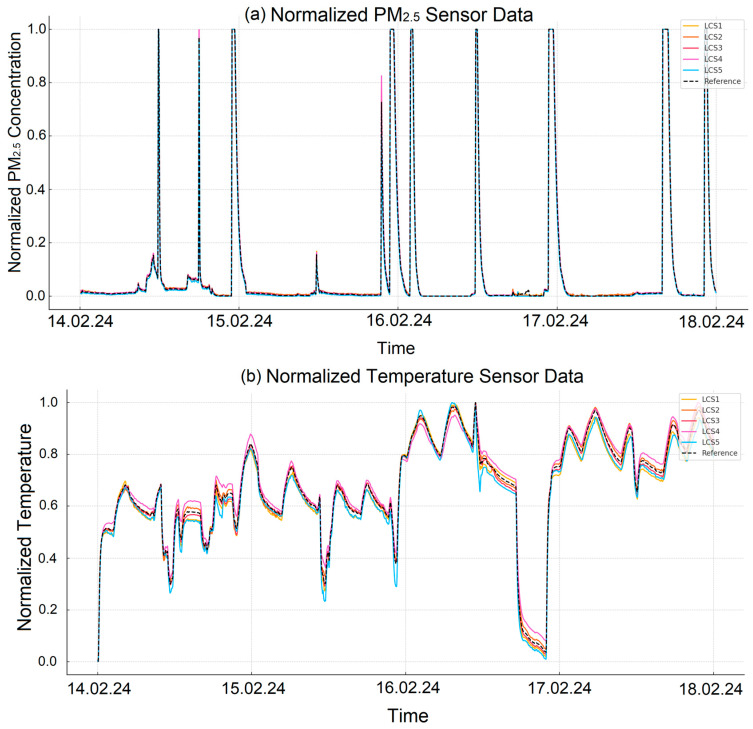

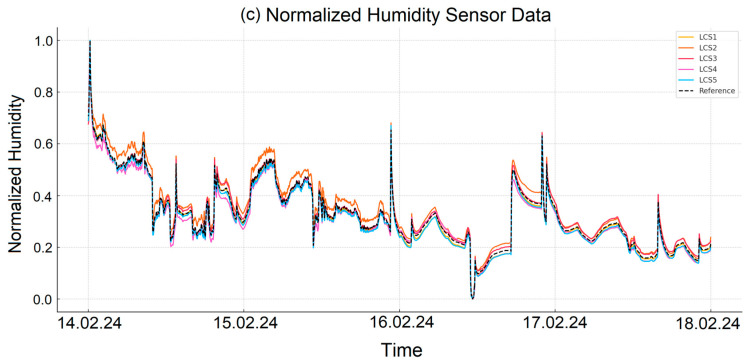
Normalized sensor data (range of 0–1): (**a**) PM_2.5_, (**b**) temperature, (**c**) humidity.

Since the raw data from the PM_2.5_, temperature, and humidity sensors did not exhibit sudden fluctuations or outliers, as observed in the CO_2_ sensors, smoothing was not applied to these sensor data. Therefore, the data were directly normalized. The graphs in Figure 2 show the normalized version of the measurements obtained from different sensors. Figure 2a shows the data obtained from the PM_2.5_ sensor normalized to the range of 0–1; Figure 2b shows the temperature sensor measurements and Figure 2c shows the humidity sensor data transformed with the same normalization method.

### 3.4. Machine Learning–Based Analysis

**Figure 3:**
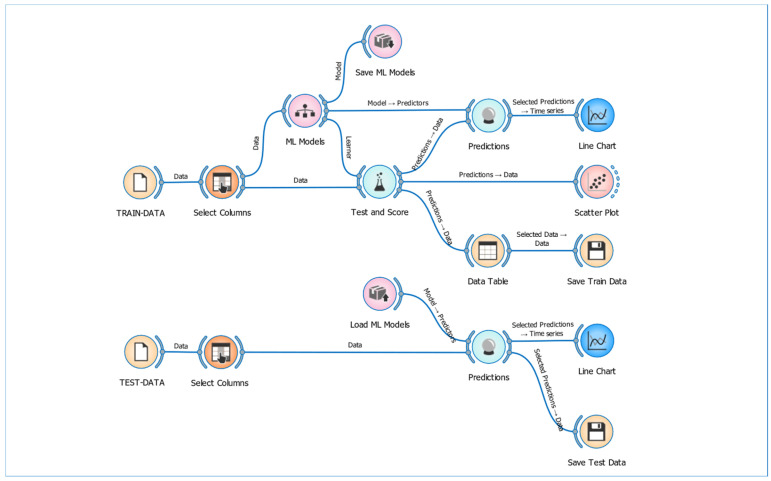
ML-based regression models and workflow for the calibration of CO_2_, PM_2.5_, temperature, and humidity sensors.

Eight different ML-based models were used for the calibration of air quality parameters. Figure 3 illustrates the workflow of regression models created in the Orange Data Mining platform for the calibration of CO_2_, PM_2.5_, temperature, and humidity sensors. This workflow was systematically constructed by organizing various widgets that include data preprocessing, model creation, evaluation, and analysis steps. The workflow starts by importing the dataset, which includes sensor measurements and reference values, into the program using the “File” widget. Next, the “Data Sample” widget is used to select random data subsets, preventing calibration models from being biased by the order of the dataset. The “Select Columns” widget is then employed to specify the columns to be used in the analysis; at this stage, the measurements from CO_2_, PM_2.5_, temperature, and humidity sensors, along with reference values, are included in the calibration process.

The main structure of the workflow consists of various ML-based regression models. These models include DT, LR, RF, kNN, AB, GB, SVM, and SGD. After completing the training and testing processes of the calibration models, the models are saved using the “Save Model” widget. This step allows the created models to be reused later or applied to different datasets. To reload the saved models, the “Load Model” widget is used, providing a time-saving advantage for future calibration processes. The “Test and Score” widget is employed to evaluate the performance of the models. This widget tests the models through methods such as cross-validation and calculates performance metrics like R^2^, RMSE, MAE, and Mean Absolute Percentage Error (MAPE). These metrics are essential for assessing the accuracy and effectiveness of each model in sensor calibration. For visualization of the calibration results, the “Line Chart” and “Scatter Plot” widgets are used. The “Line Chart” provides a graphical representation of sensor measurements, reference values, and calibrated predictions over time, allowing for a temporal analysis of model performance. On the other hand, the “Scatter Plot” widget shows the relationship between the predicted sensor values and the reference values on a two-dimensional graph. This is an essential visualization tool for evaluating the overall accuracy of the model and the success of the sensor calibration. Structured in this way, the workflow enables sensor calibration to be carried out accurately, efficiently, and comprehensively.

In this study, the selection of ML model parameters presented in Table 1 was performed through an iterative trial-and-error approach. To enhance model performance, multiple hyperparameter combinations were systematically tested for each algorithm. Throughout this process, evaluation metrics such as RMSE, MAE, and R^2^ were monitored, and the parameter configurations yielding the best overall performance and generalization capabilities were selected. Automated hyperparameter optimization techniques (e.g., grid search or Bayesian optimization) were not utilized; instead, parameters were manually tuned based on metric-based evaluation [44,45,46]. This strategy was adopted to minimize overfitting risks and ensure consistent performance across varying data samples.

### 3.5. Architecture of the IoT-Based Air Quality Monitoring System and Calibration Process

**Figure 4:**
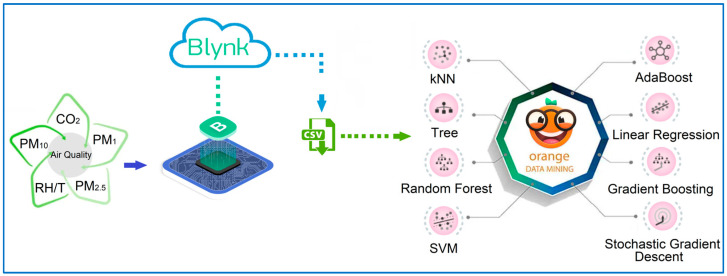
Sensor data collection and calibration process of IoT-based air quality monitoring system.

Figure 4 illustrates the architecture of the air quality monitoring and data analysis process performed using an IoT-based air quality monitoring system. The core components of the system include CO_2_, PM_2.5_, temperature, and humidity sensors, which continuously monitor air quality parameters. These sensors collect measurement data in real time. The collected data are processed by the ESP8266-12E microcontroller and transmitted to a cloud-based server through the Blynk platform at 5 s intervals. The ESP8266-12E, a 32-bit controller with an integrated WiFi module, is widely used in IoT applications [47].

The Blynk platform facilitates real-time monitoring and recording of sensor data, which are stored on the cloud in minute-averaged formats, with retrospective access available for up to six months. Additionally, the data can be downloaded in CSV format with timestamps for more detailed analysis. In the analysis phase, these recorded data are transferred to the Orange Data Mining platform, where calibration processes are performed using ML techniques. Figure 4 shows that eight different ML-based models are used in this analysis process to improve the measurement accuracy of the LCS and obtain more reliable results.

The PMS7003 is a low-cost, high-sensitivity optical PM sensor manufactured by Plantower (Beijing, China). Using laser scattering technology, it measures PM_1_, PM_2.5_, and PM_10_, and can detect particles ranging from 0.3 µm to 10 µm. It provides precise measurements within a range of 0 to 500 µg/m^3^ and updates data every second. It operates in a temperature range of −10 °C to 60 °C and a humidity range of 0% to 99% RH, transmitting digital data via the UART protocol [48]. This sensor is widely used in indoor and outdoor air quality monitoring projects, IoT applications, and smart air purifiers [49].

The MH-Z19B is a compact CO_2_ sensor operating on the non-dispersive infrared (NDIR) principle. It provides high sensitivity for CO_2_ detection and is known for its long lifespan and independence from oxygen levels. Its built-in temperature compensation feature ensures accurate measurements, and it offers flexibility with various output modes, including UART and PWM [50].

The AHT10 is a high-precision temperature and humidity sensor, offering a resolution of 0.024%RH and an accuracy of ±2%RH in humidity measurements, with long-term stability and a drift rate of <0.5%RH/year. For temperature measurements, it provides an accuracy of ±0.3 °C, operating in a range of −40 °C to 85 °C. These features make the AHT10 ideal for applications such as HVAC systems, smart home devices, and smart agriculture [51].

**Figure 5:**
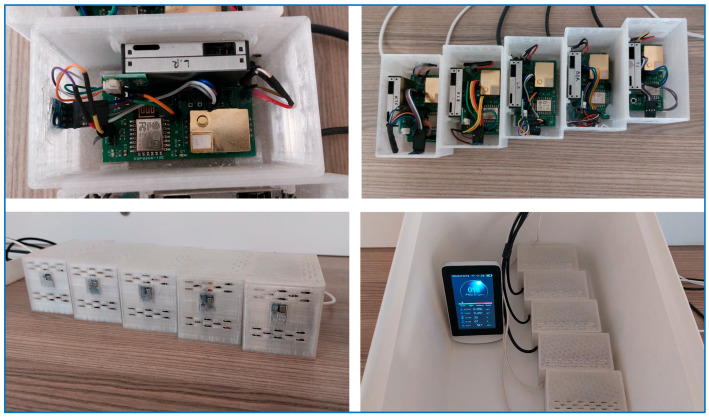
Air quality monitoring system: visual depiction of calibration chamber and sensors.

A homogeneous and controlled environment is essential for the accurate calibration of LCS. The environmental calibration chamber, depicted in Figure 5, ensures stable air distribution for the sensors, shielding them from sudden fluctuations. The airflow, regulated by vents, is slow and controlled, preventing rapid sensor responses and allowing for calibration under consistent conditions. During the calibration process, this chamber is exposed to environments containing various pollutants, with sensors subjected to controlled emissions from different sources. For example, emissions from cooking, chemical vapors from cleaning agents, perfume, and cigarette smoke common indoor pollutants are measured by the sensors. This setup facilitates calibration against various pollutants and environmental changes, ensuring accurate and reliable results for indoor air quality assessments.

This approach aligns with scientific practices that underscore the importance of precise sensor calibration in diverse real-world scenarios, ensuring accurate data collection for indoor air quality monitoring.

### 3.6. Data Analysis and Regression Models

The sensor calibration processes were conducted using Orange Data Mining, an open-source software platform. Orange is widely adopted in academic studies due to its user-friendly interface, which facilitates data analysis processes through visual programming [52,53,54]. The platform provides a wide range of algorithms and analytical tools, allowing for the execution of data analysis steps such as data loading, visualization, preprocessing, modeling, and evaluation in a modular structure using a drag-and-drop approach. Its integration with Python enables users to develop custom analyses and ML models, offering flexibility that makes Orange a powerful tool for both research and educational purposes. In sensor calibration, eight ML-based models frequently cited in the literature were applied. Each model was calibrated using different parameters for the analysis of CO_2_, PM, temperature, and humidity sensors.

DT is an algorithm that classifies or regresses by splitting data into branches. Each node examines a specific feature and splits the data accordingly. DT breaks down the dataset into smaller, more meaningful subgroups and performs branching based on criteria such as information gain or the Gini index.

LR is used to model the linear relationship between dependent and independent variables. This model analyzes linear trends in the data, aiming to minimize the error rate of the model. However, if the data are nonlinear, model performance may decline [55].

RF is an ensemble algorithm consisting of multiple DTs. These trees are trained using different subsets of data and features. For classification, a majority vote is taken, while for regression, the average value is used. RF reduces the risk of overfitting by introducing diversity and is robust against noise in the data.

kNN is a simple yet effective method used for classification and regression tasks. It identifies the k nearest neighbors in the dataset and assigns the majority class or average value of these neighbors as the predicted result.

AB is an ensemble algorithm that aims to build a strong model by sequentially combining weak learners. AB increases the weight of misclassified data in each step, allowing new learners to correct these errors, thereby improving model performance.

GB is a sequential ensemble learning algorithm designed to minimize errors by adding weak learners at each step. Each new model corrects the errors of the previous one, and this process is managed through gradient calculations. GB performs particularly well on complex datasets.

SVMs aim to find the optimal hyperplane to separate data points in classification and regression problems. If the data are not linearly separable, kernel functions are used to perform separation in a higher-dimensional space.

SGD is a method used for optimization in large datasets. SGD updates the parameters using only one data point at each step, which increases computation speed and optimizes memory usage. However, due to using only one data point, the learning process can be noisier.

### 3.7. Performance Metrics

The evaluation metrics used to assess the performance of ML models play a critical role in understanding prediction accuracy and the model’s generalization ability. These metrics enable the evaluation of the model’s performance from various perspectives by assessing its accuracy and errors [56]. They are used to evaluate the model’s performance on both training and test datasets, helping to identify its generalization ability and success across different data sets. This way, issues like overfitting can be detected, and the model’s performance on different datasets can be more clearly analyzed. To evaluate the performance of the different ML models used in sensor calibration, we employed the R^2^, MAE, RMSE, and MAPE metrics. Below, the formulas and descriptions of these metrics are provided together. R^2^ indicates how well the model fits the actual data. It is calculated using the following formula:

(1) $$R^{2}=1-\frac{\sum_{i=1}^{n}{\left(y_{i}-\hat{y_{i}}\right)}^{2}}{\sum_{i=1}^{n}{\left(y_{i}-\bar{y_{i}}\right)}^{2}}$$

In Formula (1), *yi* represents the actual values, $\hat{y_{i}}$ represents the values predicted by the model, and $\bar{y_{i}}$ represents the mean of the actual values. R^2^ ranges between 0 and 1, with values closer to 1 indicating higher prediction accuracy of the model.

MAE is a commonly used metric to evaluate the accuracy of a model’s predictions. MAE helps us understand the magnitude of errors by calculating the average of the absolute differences between the predicted values and the actual values. It is computed using the following formula:

(2) $$MAE=\frac{1}{n}\sum_{k=0}^{n}\left|y_{i}-{\hat{y}}_{i}\right|$$

In Formula (2), *yi* represents the actual values, ${\hat{y}}_{i}$ represents the predicted values, and *n* denotes the total number of data points in the dataset.

RMSE is another important error metric used to measure the magnitude of the differences between a model’s predictions and the actual values. RMSE is calculated by taking the average of the squared errors and then taking the square root of this value. This metric is represented by the following formula:

(3) $$RMSE=\sqrt{\frac{1}{n}\sum_{i=1}^{n}\;{{(y}_{i}-{\hat{y}}_{i})}^{2}}$$

In Formula (3), *yi* represents the actual values, ${\hat{y}}_{i}$ represents the predicted values, and *n* denotes the total number of data points in the dataset. RMSE is a metric that should be particularly noted when evaluating a model’s error performance, as it highlights larger errors.

MAPE is an error metric that shows how close a model’s predictions are to the actual values in percentage terms. MAPE calculates the average percentage difference between the predicted and actual values. This metric is used to understand the magnitude of prediction errors; a low MAPE indicates high model accuracy. The formula for MAPE is as follows:

(4) $$MAPE=\frac{1}{n}\sum_{i=1}^{n}\left|\frac{\left(y_{i}-{\hat{y}}_{i}\right)}{y_{i}}\right|\times 100$$

In Formula (4), *yi* represents the actual values, ${\hat{y}}_{i}$ represents the predicted values, and *n* denotes the total number of data points in the dataset. MAPE provides a percentage-based error measure; however, it can encounter sensitivity issues when actual values approach zero.

## 4. Results and Discussion

In this study, various ML models were employed for the calibration and performance evaluation of CO_2_, PM_2.5_, temperature, and humidity sensor data. These models were rigorously analyzed on both training and test datasets using performance metrics such as RMSE, MAE, MAPE, and R^2^. The success of the models in calibration was linked to low error rates and their ability to generalize to new data. Ensuring alignment between the models and reference data during the calibration process is crucial for the accuracy of sensor outputs. In this context, the performance of each model on both the training and test sets was compared, with a particular focus on their effectiveness in the test sets.

**Figure 6:**
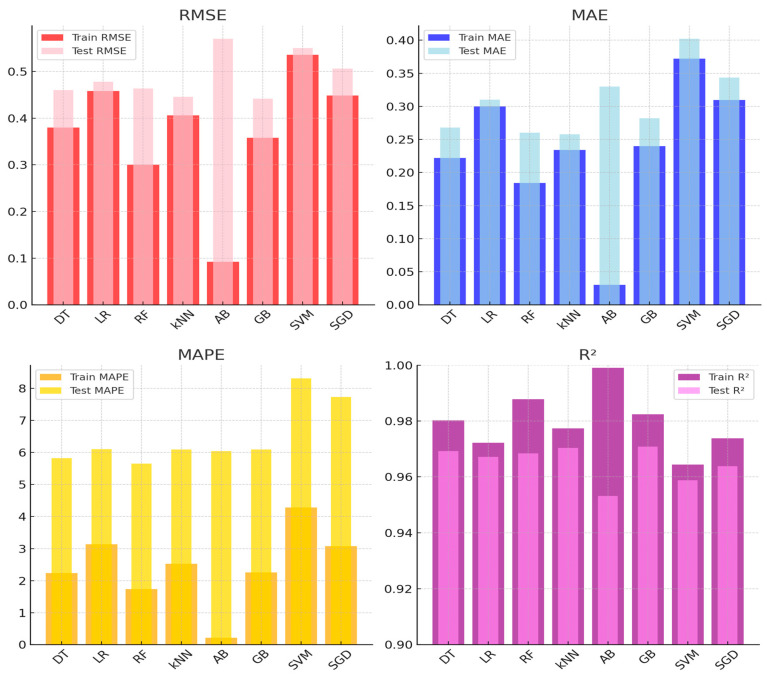
Performance evaluation of ML models for CO_2_ sensor calibration.

Figure 6 presents the analysis metrics for ML models applied to CO_2_ sensors using training and test datasets. While GB and kNN demonstrated the highest accuracy for CO_2_ sensor calibration, their computational demands were higher compared to simpler models like LR. This trade-off should be considered in resource-constrained applications.

Table 2 presents the performance metrics of ML models for CO_2_ sensors on training and test datasets. The best-performing models are GB and kNN. On the test dataset, GB achieved the highest R^2^ value (0.970) and recorded low RMSE (0.442) and MAE (0.282) values. Similarly, kNN demonstrated strong performance with a test RMSE of 0.446 and the lowest MAE (0.258), accompanied by a high R^2^ value of 0.970. On the other hand, the SVM model exhibited the lower performance among all models. It recorded the highest MAPE value (8.312) on the test dataset, along with high RMSE (0.550) and MAE (0.402) values, reflecting poor generalization to unseen data.

**Figure 7:**
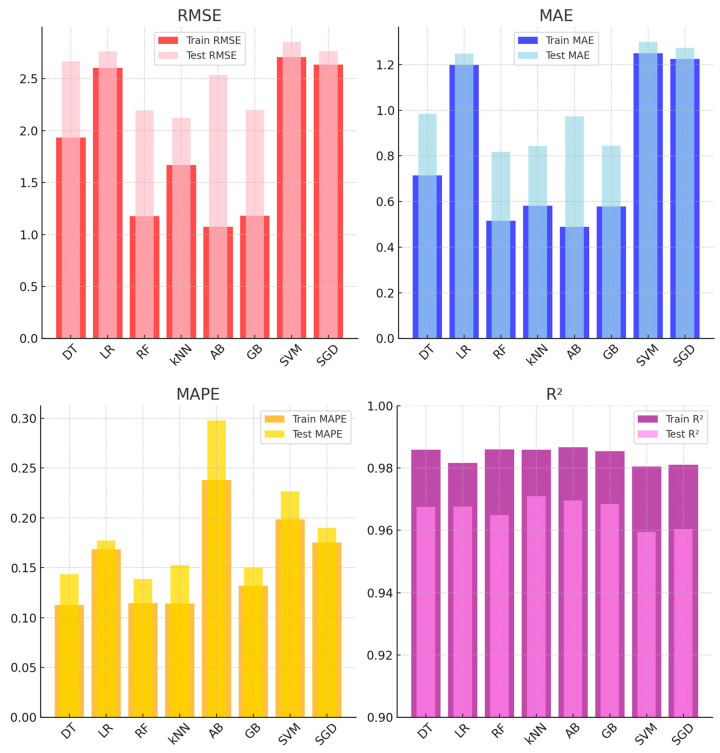
Performance evaluation of ML models for PM_2.5_ sensor calibration.

Figure 7 presents the performance metrics of ML models for PM_2.5_ sensor calibration based on training and test datasets. Among the evaluated models, kNN and RF demonstrated consistent performance, achieving low error rates and high R^2^ values in the test set. kNN achieved a test RMSE of 2.123, an MAE of 0.842, and the highest R^2^ value of 0.970, indicating reliable predictive accuracy. Similarly, RF achieved a test RMSE of 2.194, an MAE of 0.817, and an R^2^ of 0.964, showing strong generalization.

Models such as SVM and SGD recorded higher error rates in the test set. SVM had a test RMSE of 2.855 and MAE of 1.299, while SGD followed with a test RMSE of 2.764 and MAE of 1.273, suggesting challenges in maintaining accuracy across datasets. The performance of AB highlighted a notable gap between the training and test sets. While it achieved strong results during training (RMSE: 1.075, MAE: 0.489), its test performance declined, with an RMSE of 2.535 and MAE of 0.972, suggesting potential overfitting. GB, on the other hand, performed consistently, with a test RMSE of 2.198, an MAE of 0.844, and an R^2^ of 0.968. In summary, kNN and RF emerged as the most reliable models for PM_2.5_ sensor calibration, while SVM, SGD, and AB demonstrated varying degrees of difficulty in generalizing to the test data. Similarly, other studies have reported that the RF model demonstrated strong performance for PMS5003 and PMS7003 sensors, achieving R^2^ values of 0.97 and 0.92, respectively [57]. In another study focusing on the calibration of the PMS5003 sensor, the performance of nine different ML regression algorithms was evaluated. Among these algorithms, kNN and RF demonstrated superior performance, achieving test scores of 0.97 and 0.96, respectively [23].

**Figure 8:**
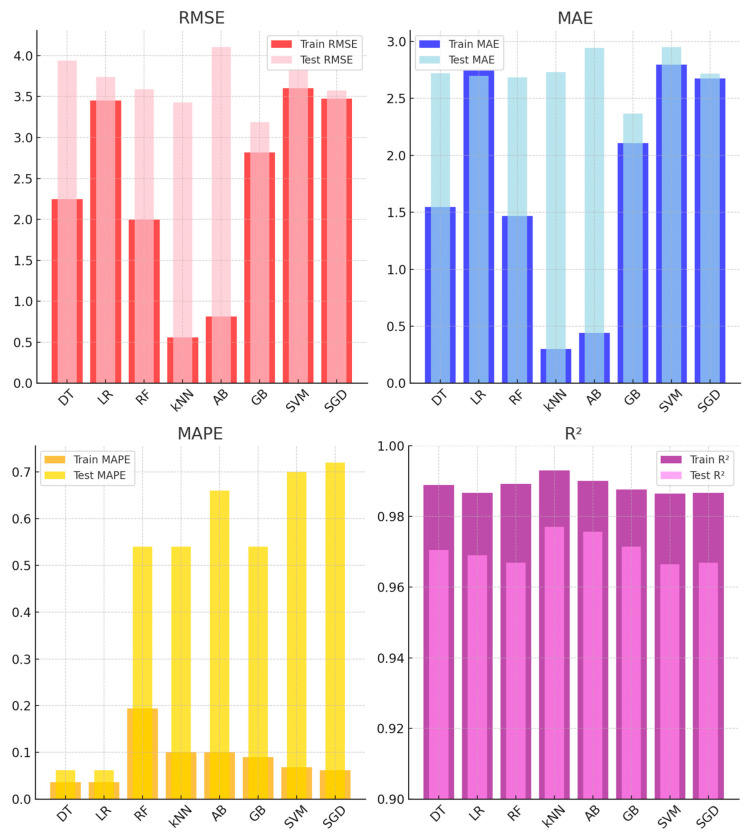
Average performance metrics of different models for humidity sensors on training and test sets.

Figure 8 shows the performance of ML models for humidity sensor calibration across training and test datasets. kNN and GB delivered the best results, with kNN achieving an R^2^ of 0.977 and a test RMSE of 3.430. GB followed with an R^2^ of 0.971 and a test RMSE of 3.188. In contrast, LR and SGD showed moderate performance. LR had a test RMSE of 3.740, while SGD recorded 3.574. AB, despite excelling in training (RMSE: 0.814), overfitted, resulting in a test RMSE of 4.106. SVM showed the highest test RMSE (3.842), indicating weak generalization. Overall, kNN and GB emerged as the most reliable models, while AB and SVM struggled to generalize effectively.

**Figure 9:**
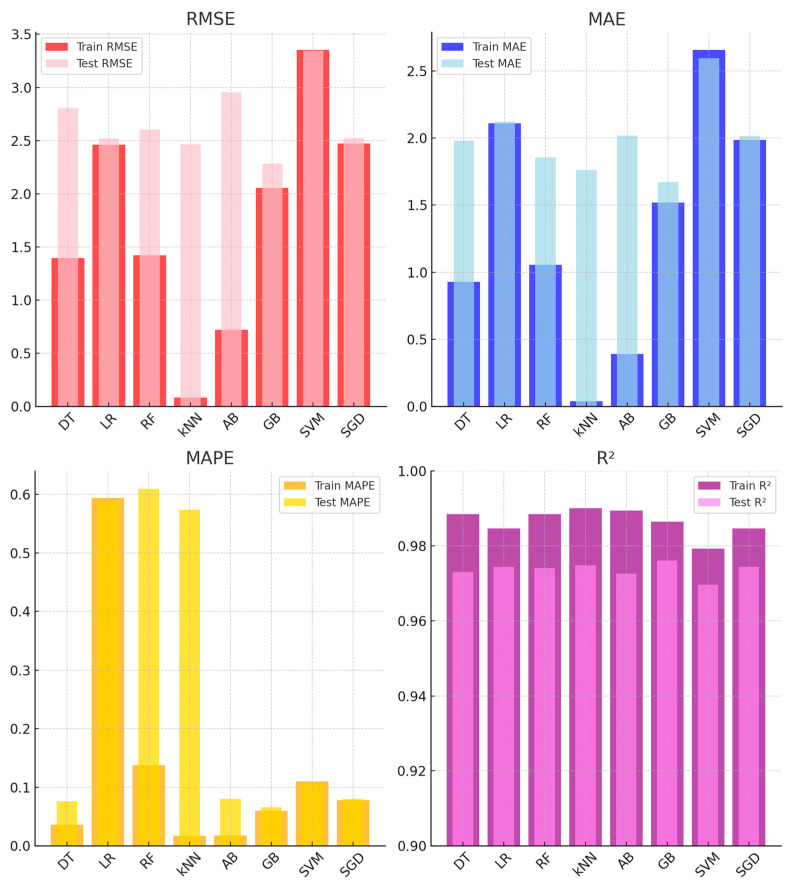
Average performance metrics of different models for temperature sensors on training and test sets.

Figure 9 presents the performance comparison of various ML models for temperature sensor calibration across training and test datasets. Among the models, GB and kNN demonstrated superior performance with low error rates and high predictive accuracy. In the test set, the GB model achieved an RMSE of 2.284, an MAE of 1.672, and an R^2^ of 0.976, while the kNN model closely followed with an RMSE of 2.466, an MAE of 1.762, and an R^2^ of 0.974. LR model provided moderate accuracy, though its MAPE (0.588) suggested relatively higher proportional error. Despite exhibiting strong performance in the training set (RMSE: 0.720, MAE: 0.390), the AB model showed a marked decline in the test set (RMSE: 2.954, MAE: 2.016). In contrast, SVM and SGD exhibited higher error rates, with the SVM model recording the largest RMSE (3.344) and MAE (2.592). Although the SGD model performed slightly better (RMSE: 2.524, MAE: 2.014), its predictive accuracy remained suboptimal. In summary, GB and kNN emerged as the most effective models for temperature sensor calibration, while SVM and AB struggled to generalize effectively to the test set.

**Figure 10:**
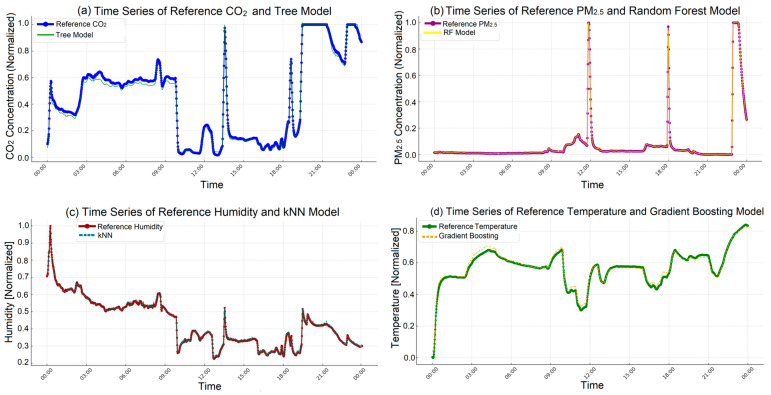
Time series comparisons of reference values and ML models for CO_2_, PM_2.5_, humidity, and temperature: (**a**) time series of reference CO_2_ and DT model, (**b**) time series of reference PM_2.5_ and RF model, (**c**) time series of reference humidity and kNN model, (**d**) time series of reference temperature and GB model.

The graphs presented in Figure 10 illustrate the calibration results of CO_2_, PM_2.5_, humidity, and temperature sensors using various ML models on the test data. In Figure 10a, it can be seen that the DT model generally aligned well with the reference CO_2_ values in the test data, although slight deviations were detected during sudden fluctuations. In Figure 10b, the RF model successfully captured long-term trends in PM_2.5_ concentrations in the test data, but some deviations from the reference data were observed during sharp increases. Figure 10c shows that the kNN model largely aligned with the reference humidity values in the test data, though delays in the model’s response were noted during sudden humidity changes. In Figure 10d, the GB model demonstrated strong alignment with the reference temperature data over long-term trends in the test data, but it responded more slowly to abrupt temperature changes.

Table 3 summarizes the performance metrics of various ML models across all sensors, providing a comprehensive evaluation of their calibration accuracy. kNN, GB, and RF consistently delivered reliable results, with relatively low error rates and high R^2^ values in the test datasets. Among these, GB achieved the lowest RMSE (2.028) and MAE (1.291) in the test set, coupled with a strong R^2^ value of 0.971, indicating robust generalization. Similarly, kNN demonstrated competitive performance with an R^2^ of 0.973 and a test RMSE of 2.116, reflecting its efficiency in sensor calibration.

In contrast, models such as LR and SGD exhibited moderate accuracy. While LR maintained an R^2^ of 0.969 and a test RMSE of 2.375, SGD showed slightly higher errors, with a test RMSE of 2.342 and an R^2^ of 0.966. The AB model performed exceptionally well during training (RMSE: 0.675, MAE: 0.337), but its test performance (RMSE: 2.541, R^2^: 0.967) revealed a tendency toward overfitting.

The SVM model consistently exhibited the highest error rates across the metrics, with a test RMSE of 2.647 and the lowest R^2^ value (0.963), indicating challenges in its ability to generalize effectively to unseen data. This suggests that SVM may not be ideal for sensor calibration tasks in this context.

In this study, the performance of eight different machine learning algorithms was found to vary significantly depending on the type of sensor used and the pollutant being measured. This variation stems primarily from the physical measurement principles of the sensors and the statistical characteristics of the data they generate. For instance, CO_2_ sensors typically produce lower-variance, more stable data, which favors models like GB that perform well on smoother distributions (Table 2, Figure 6). In contrast, PM_2.5_ sensors are more sensitive to environmental fluctuations and generate noisier data, where algorithms such as kNN and RF demonstrate superior performance (Table 3, Figure 7). Likewise, in relatively stable environmental parameters such as temperature and humidity, kNN outperformed other models with lower error rates (Figure 8 and Figure 9). These findings indicate that the optimal algorithm varies across sensor types and pollutant characteristics.

Furthermore, five sensors of each type were used in the study, which was a deliberate design choice to increase the generalizability of the findings. By deploying multiple sensors under identical environmental conditions, it was possible to assess inter-sensor variability and minimize the influence of potential manufacturing inconsistencies. Consequently, the machine learning–based calibration models developed in this study do not rely on the behavior of a single sensor but rather reflect the average performance of a sensor family. This effect is particularly evident in the time-series results presented in Figure 10, where predictions generated by the models closely follow the trends of the reference measurements. For example, the RF model accurately tracks PM_2.5_ variations (Figure 10b), while the kNN model yields consistent predictions for humidity (Figure 10c), showcasing the added reliability of using multiple sensor instances during model development.

These findings reinforce that a single algorithm may not perform optimally across all sensor types and environmental conditions. Instead, algorithm selection should be tailored to the statistical nature of the pollutant being measured. Moreover, using multiple sensors enhances model robustness and broadens the applicability of the calibration approach for large-scale air quality monitoring networks.

In conclusion, kNN, GB, and RF emerged as the most effective models for sensor calibration, demonstrating a balance between low error rates and strong generalization across multiple sensor types. These findings underscore the necessity of selecting models that are well-suited to the statistical behavior of the target pollutant and sensor characteristics, as performance varied significantly depending on both the pollutant and the algorithm used.

## 5. Conclusions

This study evaluated the effectiveness of ML-based calibration methods to enhance the applicability of LCS in indoor air quality monitoring systems. A comparative analysis of eight different ML algorithms revealed that GB and kNN models achieved the highest accuracy. For CO_2_ sensor calibration, GB achieved R^2^ = 0.970, RMSE = 0.442, and MAE = 0.282, providing the lowest error rate. For the PM_2.5_ sensor, kNN produced the most accurate results, with R^2^ = 0.970, RMSE = 2.123, and MAE = 0.842. For temperature and humidity sensors, GB demonstrated the best accuracy, with R^2^ = 0.976 and RMSE = 2.284. These findings indicate that, by identifying suitable ML methods, the accuracy of LCSs can be significantly improved. The IoT-based air quality monitoring system developed in this study was tested against common indoor pollutants such as cigarette smoke, human respiration–derived CO_2_ emissions, food vapors, and cleaning chemicals, providing real-time monitoring capability. The results demonstrate that ML-supported calibration techniques can serve as a viable alternative to high-cost air quality monitoring systems.

However, the study has some limitations. The dataset was collected in a specific indoor environment over a limited period, which restricts evaluation of long-term model performance and robustness under varying environmental conditions. Additionally, some ML models exhibited notable differences in performance between training and test datasets, indicating the need for further optimization to improve generalization capability.

Future studies should collect long-term data and test ML-based calibration under diverse environmental conditions to assess its broader applicability. Furthermore, the integration of deep learning algorithms, the combination of multiple sensor types, and the use of larger datasets could further enhance model generalization and calibration accuracy. In addition, incorporating environmental parameters such as temperature and humidity as input features in ML models could improve calibration stability by accounting for external factors affecting sensor behavior. Advancements in ML-based calibration techniques will contribute to making low-cost sensors more reliable and widely applicable in air quality monitoring systems.

## Figures and Tables

**Table 1:** Parameters of ML models used for CO_2_ sensor calibration.

Model	Parameters
DT	Min. instances: 20; Max depth: 500
RF	Trees: 50; Depth: 15; Min. split: 5
LR	Fit Intercept: Active; Regularization: None
GB	Trees: 200; L. Rate: 0.1; Depth: 5; Min. Split: 2; T. Fraction: 0.85
SVM	C: 0.90; Epsilon: 0.10; Kernel: Linear; Numerical Tolerance: 0.0200
AB	Estimators: 80; Learning Rate: 1.0; Reg. Loss Function: Linear
kNN	Neighbors: 20; Metric: Euclidean;
SGD	Loss: L. Regression: Huber, Regularization: Lasso L1, Strength: (α: 0.0040); Tolerance: 0.0013

**Table 2:** Performance metrics of ML models for CO_2_ sensors on training and test datasets.

	TRAIN				TEST			
Models	RMSE	MAE	MAPE	R^2^	RMSE	MAE	MAPE	R^2^
DT	0.380	0.222	2.234	0.980	0.460	0.268	5.820	0.969
LR	0.458	0.300	3.142	0.972	0.478	0.310	6.098	0.967
RF	0.300	0.184	1.740	0.987	0.464	0.260	5.652	0.968
kNN	0.406	0.234	2.532	0.977	0.446	0.258	6.096	0.970
AB	0.092	0.030	0.212	0.999	0.570	0.330	6.046	0.953
GB	0.358	0.240	2.258	0.982	0.442	0.282	6.092	0.970
SVM	0.536	0.372	4.280	0.964	0.550	0.402	8.312	0.958
SGD	0.448	0.309	3.080	0.973	0.506	0.343	7.735	0.963

**Table 3:** Average performance metrics of ML models across all sensors.

	TRAIN				TEST			
Models	RMSE	MAE	MAPE	R^2^	RMSE	MAE	MAPE	R^2^
DT	1.489	0.852	0.604	0.985	2.468	1.487	1.525	0.970
LR	2.243	1.587	0.985	0.981	2.375	1.594	1.731	0.969
RF	1.223	0.805	0.546	0.987	2.212	1.404	1.735	0.968
kNN	0.679	0.279	0.690	0.986	2.116	1.398	1.840	0.973
AB	0.675	0.337	0.142	0.991	2.541	1.565	1.770	0.967
GB	1.602	1.111	0.635	0.985	2.028	1.291	1.712	0.971
SVM	2.550	1.768	1.164	0.977	2.647	1.811	2.336	0.963
SGD	2.257	1.548	0.848	0.981	2.342	1.587	2.181	0.966

## Data Availability

The original contributions presented in this study are included in the article. Further inquiries can be directed to the corresponding author.
